# Balancing secondary intention and reconstruction in nail apparatus melanoma: Plastic surgery insight from a case serie

**DOI:** 10.1016/j.ijscr.2025.112030

**Published:** 2025-10-06

**Authors:** Elise Lupon, Luc Chouquet, Olivier Camuzard

**Affiliations:** a University Institute of Locomotor and Sport (IULS), Pasteur Hospital, 30 voie romaine, 06100, Nice, France; b Université Côte d'Azur, CNRS, LP2M, France

**Keywords:** Nail apparatus melanoma, Secondary intention healing, Dermal substitute, Perforator flap, Reconstructive surgery

## Abstract

• Secondary intention healing remains an option for nail apparatus melanoma.
• Healing time is long and cosmetic results can be unpredictable.
• Local flaps or dermal substitutes allow faster healing and better outcomes.
• Perforator flaps can improve comfort and long-term functional results.

Melanoma of the nail apparatus is a rare entity that often poses difficult reconstructive dilemmas, which remain relatively unfamiliar to many plastic surgeons and dermatologists given the recent interest in this topic. Publications remain scarce, and the use of validated functional outcome measures such as the QuickDASH, combined with patient-reported satisfaction, is still limited in this field [1].

Secondary intention healing has been frequently emphasized for its advantages, including technical simplicity, avoidance of donor-site morbidity, and acceptable hand function [1]. Goettmann et al. reported on 63 cases, of which 52 were treated by secondary intention, with a mean follow-up of 120 months, concluding that long-term results were satisfactory [3]. These findings reinforce that complex reconstructions are not always mandatory and that secondary healing remains a valid low-technology option, particularly in fragile patients or in settings with limited reconstructive expertise. As some authors have noted [2,3], an additional advantage lies in avoiding donor-site morbidity from one-stage reconstruction, which can be particularly useful if revision is required in R1 cases. In our practice, we generally propose one-stage reconstructions only for melanoma in situ, to minimize this risk.

However, as demonstrated in our 2025 systematic review [1], secondary healing also has limitations. Healing may extend 8–12 weeks, delaying rehabilitation and return to work [2]. Cosmetic outcomes are unpredictable, with possible dyschromia, hypersensitivity, or keratinized spicules impairing satisfaction despite preserved function, especially when bone exposure is left to granulate [4]. Moreover, 17 % of patients in one series reported intense postoperative pain [2]. Several reconstructive techniques can now provide alternatives to secondary intention, offering faster healing, sometimes in just 2 weeks, and more predictable outcomes [1]. For instance, dermal substitutes combined with skin grafts have been shown to reduce bone hypersensitivity, improve cosmetic integration [1,4,5], enable a more aggressive approach to melanoma excision [5], and limit retraction effects, as illustrated in the case shown in Fig. 1, reported in accordance with the SCARE guidelines [6]. Local advancement or propeller flaps can also provide rapid coverage with satisfactory color match and thickness, thereby reducing healing time and improving patient comfort, as illustrated by our one-year follow-up of a first dorsal metatarsal artery perforator flap case (Fig. 2.) [7]. These approaches are particularly relevant in younger or active patients, for whom function and cosmesis are paramount.

**Fig. 1:**
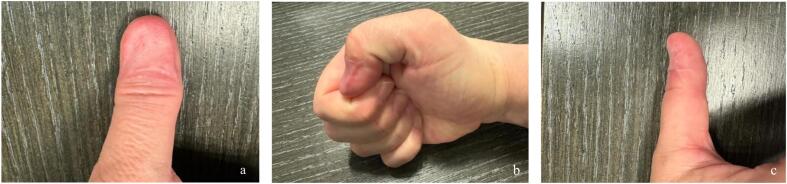
One-year postoperative views of the thumb in a patient with in situ subungual melanoma treated by wide excision and reconstructed with dermal matrices and a split-thickness skin grafts, showing minimal retraction and preserved function. (a) Dorsal aspect. (b) Radial aspect. (c) Ulnar aspect.

**Fig. 2:**
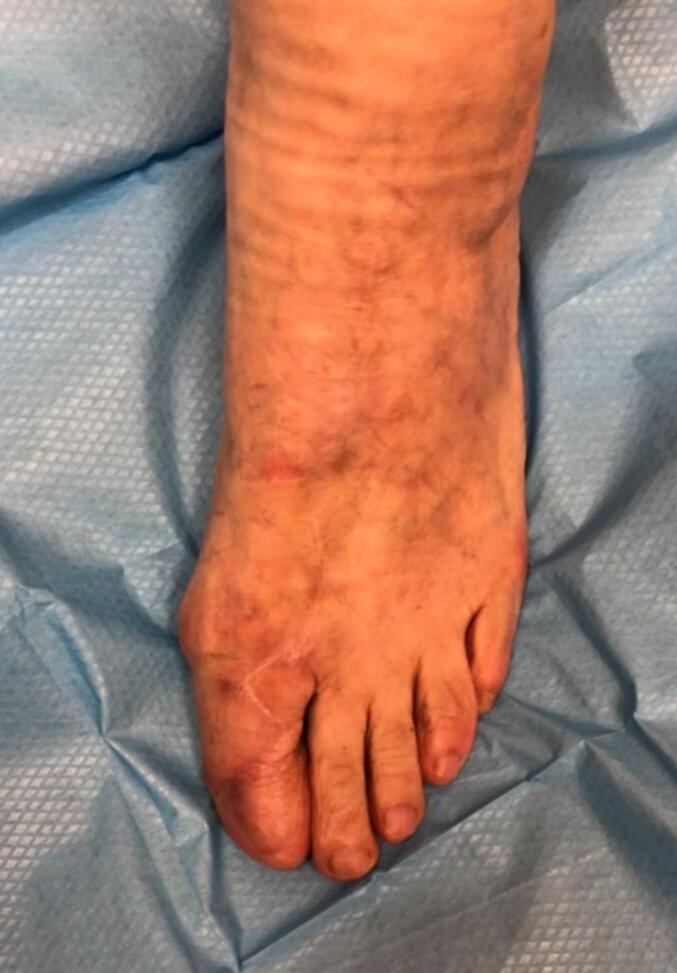
Postoperative view at 16 months of a 74-year-old female treated for acro-lentiginous melanoma of the left hallux (pTis). Coverage was achieved using a first dorsal metatarsal artery perforator flap, with the donor site closed using Integra™. The long-term outcome shows stable coverage, good color match, and satisfactory functional and cosmetic results.

Another important consideration concerns the Breslow threshold for wide local excision (WLE) and conservative treatment. Based on our dermatology team's experience, and in line with some authors [1,2], we use a cutoff of ≤0.5 mm [2]. However, the literature remains ambiguous, with an increasing number of authors advocating for a more tolerant threshold of 0.8 mm [2]. Given the importance of maximizing conservative options, we encourage re-analysis of recurrence rates with longer follow-up to refine this cutoff.

In conclusion, we believe the trend toward secondary intention healing in nail apparatus melanoma should be nuanced. While it may be appropriate in resource-limited settings or for fragile patients, reconstructive options should be prioritized in younger or more active patients. Collaboration between dermatologists and reconstructive plastic surgeons is essential to optimize outcomes. Future progress will require a clearer definition of the Breslow cutoff for WLE (0.5 vs. 0.8 mm) and larger, prospective, multicenter studies to refine oncologic and reconstructive algorithms for nail apparatus melanoma.

## Consent

All patients had signed informed consent for anonymous use of their data and for body donation to science while they were alive.

## Ethical statement

The local Ethical Committee and the Institutional Review Board approved this study.

## Guarantor

Dr. Elise Lupon and Prof Olivier Camuzard accept full responsibility for the work, have access to the patient's information, and decide to publish.

## SCARE guideline

The work has been reported in line with the SCARE criteria 2025.

## Sources of funding

None. This research received no specific grant from any funding agency in the public, commercial, or not-for-profit sectors.

## Author contribution

E. Lupon conceived the study, wrote the first draft of the manuscript, and reviewed the final version.

L. Chouquet curated and collected the data, and reviewed the manuscript.

O. Camuzard supervised the project and critically reviewed the manuscript.

## Declaration of competing interest

The authors declare no funding and no conflict of interest related to this work.
